# HER2 Status in Gastric and Gastroesophageal Junction Cancer Assessed by Local and Central Laboratories: Chinese Results of the HER-EAGLE Study

**DOI:** 10.1371/journal.pone.0080290

**Published:** 2013-11-14

**Authors:** Dan Huang, Ning Lu, Qinhe Fan, Weiqi Sheng, Hong Bu, Xiaolong Jin, Guimei Li, Yanhui Liu, Xianghong Li, Wenyong Sun, Huizhong Zhang, Xiaobing Li, Zongguang Zhou, Min Yan, Xuan Wang, Weihong Sha, Jiafu Ji, Xiangdong Cheng, Zhiwei Zhou, Jianming Xu, Xiang Du

**Affiliations:** 1 Department of Pathology, Cancer Center of Fudan University; Department of Oncology, Shanghai Medical College, Fudan University, Shanghai, China; 2 Department of Pathology, Cancer Institute and Hospital, Chinese Academy of Medical Sciences, Beijing, China; 3 Department of Pathology, Jiangsu Province Hospital, Nanjing, China; 4 Department of Pathology, West China Hospital of Sichuan University, Chengdu, China; 5 Department of Pathology, Shanghai Jiao Tong University School of Medicine Ruijing Hospital, Shanghai, China; 6 Department of Pathology, PLA 81 Hospital, Nanjing, China; 7 Department of Pathology, Guangdong General Hospital, Guangzhou, China; 8 Department of Pathology, Beijing Cancer Hospital, Beijing, China; 9 Department of Pathology, Zhejiang Cancer Hospital, Hangzhou, China; 10 Department of Pathology, Sun Yat-sen University Cancer Center, Guangzhou, China; 11 Department of Pathology, PLA 307 Hospital, Beijing, China; 12 Center of Gastrointestinal Surgery, West China Hospital of Sichuan University, Chengdu, China; 13 Department of Surgery and Shanghai Institute of Digestive Surgery, Shanghai Jiao Tong University School of Medicine Ruijing Hospital, Shanghai, China; 14 Department of Surgery, PLA 81 Hospital, Nanjing, China; 15 Department of Gastroenterology, Guangdong General Hospital, Guangzhou, China; 16 Department of Gastrointestinal Surgery, Beijing Cancer Hospital, Beijing, China; 17 Department of Abdominal Surgery, Zhejiang Cancer Hospital, Hangzhou, China; 18 Department of Gastric and Pancreatic Surgery, Sun Yat-sen University Cancer Center, Guangzhou, China; 19 Department of Gastrointestinal Oncology, PLA 307 Hospital, Beijing, China;; The Chinese University of Hong Kong, Hong Kong

## Abstract

Trastuzumab has been approved for human epidermal growth factor receptor 2 (HER2)-positive advanced gastric and gastroesophageal junction cancers (GC and GJC) in combination with chemotherapy. The aim of this HER2 early/advanced gastric epidemiology (HER-EAGLE) study was to evaluate the frequency of HER2 over-expression and to evaluate agreement on HER2 status assessment in GC and GJC patients in local laboratories versus a central laboratory in China. Tumor samples from 734 GC or GJC patients who were enrolled at 11 different hospitals in China were examined. HER2 status was assessed by immunohistochemistry (IHC), and followed by dual-color silver-enhanced in Situ hybridization (DSISH) in IHC 2+ cases. Clinicopathologic characteristics were collected from all of the patients. HER2-positive tumors were identified in 12.0% (88/734) of the GC and GJC cases. There were significantly higher rates of HER2 positivity in patients with GJC (GJC: 18.1%, GC: 9.7%, *P*=0.002), and intestinal-type cancers using the Lauren classification (intestinal: 23.6%, diffuse/mixed: 4.3%, *P*<0.0001). No significant difference in HER2 positivity was identified between resection and biopsy samples, or between early and advanced disease stages. The agreement between local laboratories and the central laboratory on HER2 status scoring was good (kappa=0.86). The main reason of HER2 status discordance between local and the central laboratories was IHC result mis-interpretation in local laboratories. These results suggest that IHC followed by DSISH testing is an accurate and cost-effective procedure in China.

## Introduction

Gastric cancer (GC) and gastroesophageal junction cancer (GJC) are the second most prevalent causes of cancer-related mortality worldwide [1]. Although the incidence and mortality of GC have been slowly decreasing over the past several decades [2,3], the disease remains the third most common cancer in China, with 400,000 new cases and 300,000 estimated deaths annually [4]. Recently, studies have demonstrated the rapid rise in the incidence of GJC in many countries [5-7]. The majorities of patients with GC or GJC are diagnosed at an advanced, metastatic or inoperable stage and have poor outcomes. The five-year overall survival rate for advanced GC or GJC is approximately 5–20% [8,9]. Chemotherapy can modestly improve overall survival rate, however, there is no single well-established standard of care for metastatic GC or GJC. 

Given the need for more efficacious treatment for metastatic GC and GJC, molecularly targeted therapies were under investigation. Among these is trastuzumab, a monoclonal antibody that acts against human epidermal growth factor receptor 2 (HER2), which is an effective and well-tolerated treatment for HER2-positive GC and GJC. In the ad-hoc analysis of the phase III Trastuzumab for Gastric Cancer (ToGA) study, GC or GJC patients with HER2 IHC 2+/in situ hybridization positivity, or IHC 3+, achieved the greatest OS benefit (16.0 vs. 11.8 months) [8]. The histological differences between GC and GJC, and breast cancer, including the frequent heterogeneity of HER2 over-expression, have resulted in the development of different immunohistochemistry (IHC) scoring criteria for GC and GJC, which were refined during the ToGA study [10-12]. Study of nationwide HER2 positivity rates of GC patients based on these scoring criteria in China is limited. Recently we studied HER2 positivity of samples from four medical centers [13]. However, overall consistency of HER2 status assessment between laboratories was not monitored. Considering the importance of quality control of pathologic laboratories, we sought to assess the accuracy and reproducibility of HER2 testing between laboratories by a double-blind study, and to explore potential common problems of HER2 testing in pathologic laboratories. In China, only one laboratory (Fudan University Cancer Center) has taken part in the ToGA trial and has been trained in HER2 status assessment for GC or GJC according to the update ToGA criteria. Therefore, the Fudan University Cancer Center laboratory was appointed as the central laboratory, which retested all of the tissue samples from local laboratories. The results from the local laboratories and the central laboratory were then compared.

During the process of HER2 status assessment in GCs, samples were first assessed by immunohistochemistry, with in situ hybridization to retest IHC 2+ samples [10,14]. The frequent heterogeneity in GCs can make reading fluorescence in situ hybridization (FISH) slides challenging. In this study, novel, fully automated, bright-field, dual-color silver-enhanced in-situ hybridization (DSISH) method was used because bright-field automated ISH is considered superior to FISH for determining HER2 status in samples from patients with GC [14,15]. 

The purpose of the HER2 early/advanced gastric epidemiology (HER-EAGLE) study in Chinese population was to assess the incidence rate of HER2 positivity in GC and GJC samples that were examined by local laboratories in China and to examine the inter-laboratory reproducibility between 11 local laboratories and one central laboratory. Furthermore, we explored the relationship between HER2 overexpression and clinicopathological features, and common problems causing discordance of HER2 status assessment between laboratories.

## Materials and Methods

### Ethics statement

The study was performed in accordance with local ethical and legal requirements after approval by the Ethics Committee of Fudan University Cancer Center. All participants gave written informed consent.

### Study design

The HER-EAGLE study in Chinese population was a multicenter, double-blind, comparative epidemiologic study conducted in 11 local laboratories and 1 central laboratory. In this study tumor samples and clinical data from patients with GC and GJC [16], independent of tumor stage, were reviewed. The samples were obtained from May 2011 to January 2012. In local laboratories, formalin-fixed paraffin-embedded (FFPE) tumor samples from 734 patients were assessed for HER2 status using IHC and, if a confirmatory ISH result was required (in cases of IHC 2+), a fully automated bright-field DSISH assay was performed. The cases with IHC 3+ or IHC 2+/DSISH+ were considered HER2 positive, while ones with IHC 0, IHC 1+, and IHC 2+/DSISH- were considered HER2 negative. The GC scoring criteria derived from the ToGA trial were used in this study to evaluate HER2 protein expression and gene amplification [10-12]. 721 of 734 samples examined in local laboratories were re-tested in the central laboratory (Fudan University Shanghai Cancer Center). Subsequent cuts of the same tumor blocks used in local laboratories were tested with IHC and DSISH (if IHC 2+). Thirteen cases could not be re-tested in the central laboratory, including 5 without available tumor slides and 8 with inadequate tissue samples. The results, including the clinicalpathologic characteristics, IHC results, DSISH values and final diagnosis of HER2 status, were respectively uploaded from local and central laboratories to a server. These data were blinded until all of the IHC and DSISH assays were finished in both local and central laboratories. Among the HER2 status discordant cases, if the slide used for DSISH assay in the central laboratory was available, the cases conferring IHC 2+ in the central laboratory’s examination were assessed with a dual-color, dual-hapten in-situ hybridization (DDISH) [17], a new alternative of DSISH.

### Immunohistochemistry

In both local and central laboratories, the IHC assay for HER2 protein expression was performed using the fully automated BenchMark ULTRA platform (Ventana Medical Systems, Inc., Tucson, Arizona, USA) using the Pathway anti-HER2/neu (4B5) antibody (Ventana Medical Systems, Inc., Tucson, Arizona, USA). Positive controls included breast cancer tissue with high HER2 expression. The slides were examined and scored by at least two experienced pathologists or in their group meeting in every laboratory based on previously defined scoring guidelines from the ToGA trial [10]. HER2 expression was graded using a score of 0 to 3. IHC 2+ scored tumor samples were further examined with DSISH assay.

### Dual-color silver-enhanced in-situ hybridization

Automated DSISH was performed in both local and central laboratories with the INFORM HER2 DNA Probe and the INFORM Chromosome 17 Probe on a Ventana Benchmark XT (Ventana Medical Systems). The entire assay process (including deparaffinization, pretreatment, hybridization, stringency wash, signal detection and counterstaining) was fully automated. The HER2 and chromosome 17 probes were visualized on the same slide. As a new generation of DSISH, DDISH assay has been developed to produce better signal-to-noise patterns. For gene amplification assessment, the total number of HER2 and chromosome 17 signals was counted in at least 20 tumor cell nuclei in two different areas. The HER2/chromosome 17 ratios were interpreted in accordance with the ToGA ISH scoring scheme [10].

### Statistical analysis

The data were analyzed using the Statistical Package for Social Sciences (SPSS), Version 20 (IBM, USA). A chi-squared test was used to test the association between HER2 expression and pathologic variables. To assess the multivariate analyses of the association of HER2 status with the clinicopathologic features, a logistic regression was applied. The variability between the local versus central laboratories was measured using the kappa index. All kappa indices were presented with 95% CI. All of the P values were two-sided, with a *P* value <0.05 indicating statistical significance.

## Results

### Patient and tumor characteristics

The main patient characteristics are summarized in Table 1. The patients had an average age of 60.4 years and were predominantly male (71.8%), with similar numbers of different Lauren’s tumor types (intestinal: 39.8%; diffuse: 34.6%; mixed: 25.6%). Most tumor samples were obtained from surgical resections (94.4%) and most tumors were located in the middle to distal stomach (72.9%). The TNM staging classification (American Joint Committee on Cancer, 7^th^, 2010) [18] of all of the tumors indicated that 58.3% were Stage I-IIIa, 25.5% were Stage IIIb/IIIc and 11.2% were Stage IV. 

**Table 1:** Clinicopathologic features of the study cases.

**Characteristics**		**Local Lab (n=734), n (%)**
**Age**	Median±SD	60.4±11.50
	<60	337(46.0)
	≥60	395(54.0)
	NA	2
**Gender**	Male	525(71.8)
	Female	206(28.2)
	NA	3
**Tissue type**	Resection	693(94.4)
	Biopsy	41(5.6)
Stage^*^	I-IIIa	428(58.3)
	IIIb/IIIc	187(25.5)
	IV	82(11.2)
	NA	37(5.0)
**Tumor location**	Stomach	535(72.9)
	GE junction	199(27.1)
	Intestinal	292(39.8)
**Lauren**	Diffuse	254(34.6)
	Mixed	188(25.6)

GE junction, gastroesophageal junction; NA, not available.

* Tumor stage was based on the *TNM 7* ^*th*^ *classification of tumors of the stomach*. Advanced or inoperative gastric cancer was categorized as IIIb/IIIc or IV.

### Local HER2-positivity and clinicopathologic features

A total of 734 local cases were available for analysis using IHC. All 93 IHC 2+ cases were further analyzed by DSISH. Using the IHC method, 78.6% (577/734) of the local cases were scored 0/1+, 12.7% (93/734) were scored 2+, and 8.7% (64/734) were scored 3+. About 25.8% IHC 2+ cases (24/93) were HER2-amplified as examined by DSISH assay. According to the ToGA trial ad-hoc analysis criteria (IHC 3+ or IHC 2+/ISH-amplified), 88 of 734 patients (12.0%) were diagnosed as HER2-positive in local laboratories. The association of HER2 positivity and clinicopathologic characteristics is summarized in Table 2. In the univariate analysis, HER2 positivity was more frequently detected in patients with GJC than patients with GC (18.1% vs. 9.1%, *P* = 0.002). HER2-positive tumors were also more frequently observed in intestinal tumors than in diffuse/mixed type tumors (23.6% vs. 4.3%, *P* < 0.0001). Male patients had a significantly higher incidence of HER2-positivity than female patients (14.1% (95%CI: 11.2-17.4%) vs. 6.3% (95%CI: 3.4-10.5%), *P* = 0.002) and a trend was observed between advanced age and HER2-positive tumors (*P* = 0.051), but no correlation was noted between HER2 positivity and TNM stage or specimen type (from biopsy or surgical resection). In a multivariate logistic regression analysis, HER2 positivity was only found to be statistically significantly associated with intestinal type tumors (*P* < 0.0001) and not other tumor types. 

**Table 2:** Associations between HER2 status and the clinicopathologic variables.

		**Total, n**	**No. of HER2 positivity^#^, n**	**HER2 positivity rate, (95% CI)**	**P univariate**	**P multivariate**
**Age**	<60	337	32	9.5(6.6-13.1)	0.051	0.651
	≥60	395	55	13.9(10.7-17.7)		
	NA	2				
**Gender**	Male	525	74	14.1(11.2-17.4)	0.002	0.115
	Female	206	13	6.3(3.4-10.5)		
	NA	3				
**Tumor location**	Gastric	535	52	9.7(7.3-12.6)	0.002	0.089
	GE junction	199	36	18.1(13.0-24.2)		
Stage^*^	I-IIIa	428	54	12.6(9.6-16.1)	0.379	0.338
	IIIb/c +IV	269	28	10.4(7.0-14.7)		
	NA	37				
**Lauren**	Intestinal	292	69	23.6(18.9-28.9)	<0.0001	<0.0001
	Diffuse/mixed	442	19	4.3(2.6-6.6)		
**Specimen type**	Resection	693	84	12.1(9.8-14.8)	0.441	-
	Biopsy	41	4	9.8(2.7-23.1%)		

# HER2 positivity defined as IHC 3+ or IHC 2+ /DSISH-amplified

* Tumor stage was based on the *TNM 7* ^*th*^ *classification of tumors of the stomach*. Advanced or inoperative gastric cancer was categorized as IIIb/IIIc or IV.

### Local laboratories versus central laboratory in HER2 status

**Figure 1:**
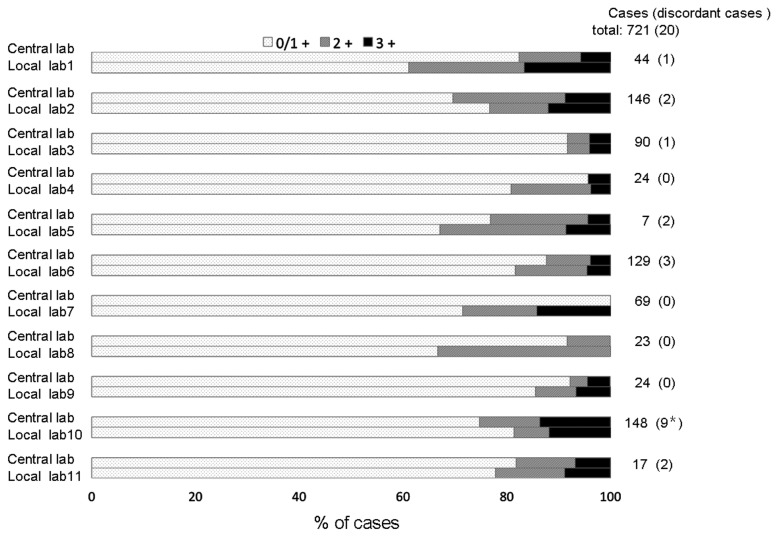
Concordance of IHC-HER2 expression between local laboratories and the central laboratory.* including the case with the manual input error.

A total of 734 samples were examined for HER2 status in local laboratories, and 721 samples were re-tested in the central laboratory. The comparative results shown in Table 3 demonstrate that the concordance of HER2 status: the total percentage agreement rate between local and central laboratories is 97.2% (kappa value 0.86, 95% CI, 0.80–0.92). The HER2-IHC comparisons are illustrated in Figure 1, showing 86.7% of agreement (kappa value 0.71, 95% CI, 0.65–0.77) between the local and central laboratories. Among the HER2 status discordant cases (Table 4), except one case with input error, the majority (14/19) were attributed to IHC discordances including 5 cytoplasmic staining cases and 2 foci strong staining (<10% of tumor) cases misdiagnosed as HER2-overexpressed (IHC 2+/3+) in local laboratories. Among the other 5 discordant DSISH results with IHC 2+ (case no. 5-7,9,10 in Table 4), 3 cases were attributed to the equivocal status of HER2 gene:chromosome 17 ratio (1.8-2.2). The discordant samples with IHC 2+ from the central laboratory, if having enough slides left in central laboratory from subsequent cuts of same tumor block used in local laboratories, were subject to DDISH assays (Table 4).

**Table 3:** Comparison of local and central laboratory HER2 testing.

	**Central Lab**, n						
**Local Labs**, n	**IHC 0/1+**, 584	**IHC 2+**, 86	**IHC 3+**, 51	**HER2 Pos**, 78	**HER2 Neg**, 643	**Agreement rate**	**Kappa values** (**95% CI**)
**IHC 0/1+**, 566	534	32	0	-	-	86.7%	0.71 (0.65-0.77)	
**IHC 2+**, 91	47	42	2	-	-			
**IHC 3+**, 64	3	12	49	-	-			
**HER2 POS**, 86	-	-	-	72	14	97.2%	0.86 (0.80-0.92)	
**HER2 NEG**, 635	-	-	-	6	629			

IHC, immunohistochemistry; CI, confidence interval; HER2 POS, HER2-positive, defined as IHC 3+ or IHC 2+/ISH-amplified; HER2 NEG, HER2-negative.

**Table 4:** Characteristics and HER2 results of the discordant slides.

**No**	**Local Lab**			**Center Lab**			**Central Review by DDISH**	
	**HER2 Status**	**IHC**	**HER2/ CEP 17 ratio**	**HER2 Status**	**IHC**	**HER2/CEP 17 ratio**	**HER2 Status**	**HER2 /CEP 17 ratio**
1	NEG	0	NA	POS	2+	2.10	POS	2.27
2	NEG	1+	NA	POS	2+	5.1^*^	FAIL	-
3	NEG	1+	NA	POS	2+	2.1	NA^∞^	
4	NEG	1+	NA	POS^#^	2+	1.8	NA^∞^	
5	NEG	2+	1.66	POS	2+	2.75	POS	2.49
6	NEG	2+	1.20	POS	2+	2.00	NA^∞^	
7	POS	2+	2.30	NEG	2+	1.84	POS	2.07
8^##^	POS	2+	5.00	NEG	0	NA	NEG	1.00
9^&^	POS	2+	2.40	NEG	2+	1.60	FAIL	-
10	POS	2+	2.52	NEG	2+	1.83	NA^∞^	
11^**^	POS	2+	2.63	NEG	0	NA	NA	
12^##^	POS	2+	2.32	NEG	0	NA	NA^∞^	
13^##^	POS	2+	2.10	NEG	1+	NA	NA^∞^	
14	POS	2+	4.18	NEG	0	NA	NA^∞^	
15	POS	2+	3.22	NEG	1+	NA	NA^∞^	
16	POS	3+	NA	NEG	2+	1.83	NEG	1.95
17	POS	3+	NA	NEG	2+	1.96	NEG	1.81
18^##^	POS	3+	NA	NEG	1+	NA	NA	
19^**^	POS	3+	NA	NEG	1+	NA	NA	
20^##^	POS	3+	NA	NEG	1+	NA	NA	

DDISH, dual-color dual-hapten in situ hybridization; IHC, immunohistochemistry; CEP17 indicates chromosome 17 centromere; POS, HER2-positive; NEG, HER2-negative; NA, not available.

& A case of CEP17 polysomy (CEP17 copy number: 3.8)

∞ Slide not available in central laboratory for DDISH testing

* A case of CEP17 monosomy (CEP17 copy number: 1.0)

# A case of manual input error during data upload

** Strongly cell membrane stained foci in <10% of tumor

# # Cytoplasmic staining

## Discussion

Recent reports from the ToGA trial have shown that HER2 status (IHC 3+ or IHC 2+/ISH+) is a predictive factor of response to trastuzumab treatment in advanced GC and GJC patients [8,19]. Thus, evaluation of HER2 status, not only for breast cancers but also for GC and GJC, is an important issue. Therefore, it is critical to assess the accuracy and reproducibility of HER2 testing between different laboratories. As a GC and GJC high-incidence country, we conducted a multicenter epidemiologic study (HER-EAGLE study) in 11 laboratories in China to evaluate the HER2 status in patients with GC or GJC. In addition, the results from local laboratories were compared with data from the central laboratory, which participated in HER2 assessment in the ToGA trial, to explore potential problems of HER2 assessment in local laboratories.

This study examined HER2 expression in 734 patients with GC or GJC from 11 hospitals. HER2 positivity was defined as IHC 3+, or IHC 2+ plus DSISH+, as per previous studies [10,12]. The proportion of HER2-positive cases in 11 local laboratories was 12.0%, which is similar to the positivity rate (13%) of another Chinese study [13]. However, it is lower than those of the ToGA study (IHC 3+ or IHC 2+/FISH+: 16.0%) and the Spanish results [20] of the HER-EAGLE study (14.1%). These apparent discrepancies between Chinese studies and other two studies may be due to differences in patient characteristics, in particular the Lauren classification and the location of the tumor. A higher percentage of intestinal-type tumors were analyzed in the ToGA study (51.4%) and the Spanish HER-EAGLE study (58.2%), while this tumor type was detected in only 39.8% of Chinese HER-EAGLE patients and 41.5% in the other Chinese study. Moreover, population in China had a lower proportion of patients with GJC (18.1% in this study and 26.5% in the other Chinese study), which possesses higher HER2 positivity rate, when compared to the ToGA study (33.2%). 

Consistent with previous studies [21-23], our results indicated that intestinal-type tumors (by Lauren classification) have higher HER2-positive ratio than tumors of the diffuse or mixed-type. Although the difference between GEJ and the distal gastric cancer did not reach statistical significance, the number of patients with GEJ tumors was small which may interfere with the power of analysis of this variable. The same may be valid for tissue source (samples from biopsy or resection), thus further studies are required to determine the appropriate quantity and location of biopsy specimens that should be obtained for accurate HER2 status assessment. 

Our data has demonstrated good overall agreement between the results from local HER2 testing and central testing (kappa=0.86, total percentage agreement rate=97.2%) mirroring a similar study for breast cancer [24]. Of note, the inter-laboratory (local vs. central) IHC agreement was slightly lower than previously observed [24] (kappa=0.71, total percentage agreement rate=86.7%). The majority of the discordant cases (14/19) were associated with incorrect IHC scoring that was observed in 7 of 11 local laboratories (Figure 1). Among these 14 IHC discordant cases, 5 cases were misdiagnosed as HER2 positive due to cytoplasmic staining and 2 cases due to strong focal staining in < 10% of tumor in local laboratories. Some factors that may contribute to this discordance include deviation from standardized testing techniques, and inexperience of local pathologists with HER2-IHC scoring for GC, implying the importance of establishing standardized laboratories and quality control system, especially for IHC, in China and other developing countries. This observation suggests that more attentions should be directed to IHC assessment training to pathologists. Different from most of the developed countries, the cost of FISH/DSISH was not covered by basic insurance/healthcare benefits in China, thus assessment of HER2 status of GC patients by IHC, followed by FISH/DSISH testing in IHC 2+ cases, is the most cost-effective and common clinical practice to identify HER2-positive patients. In fact, such misdiagnoses, especially for IHC assay, could be avoided by providing HER2 assessment training courses to pathologists to learn the unique criteria and required techniques for assessing HER2 status for GC or GJC according to the recommended guidelines [10-12]. This is a necessary and critical step to achieve the goal of providing accurate, reproducible, and reliable HER2 testing results to identify patients that potentially could benefit from trastuzumab treatment.

Our results show that the HER2 positivity rate was 12.0% in GC and GJC patients from 11 hospitals in China. A correlation between HER2 expression and Lauren classification was revealed. Good agreement on HER2 status assessment was obtained between the central and local laboratories in this study. The scoring of IHC was the largest contributor to the discordance between the 11 local laboratories and the central laboratory.
